# Identification of METTL14 in Kidney Renal Clear Cell Carcinoma Using Bioinformatics Analysis

**DOI:** 10.1155/2019/5648783

**Published:** 2019-12-30

**Authors:** Qian Wang, Hao Zhang, Quanbing Chen, Zhenghua Wan, Xiaoyong Gao, Wenhui Qian

**Affiliations:** ^1^Department of Oncology Surgery, Nanjing First Hospital, Nanjing Medical University, Nanjing, China; ^2^Department of Nephrology, Nanjing First Hospital, Nanjing Medical University, Nanjing, China; ^3^Department of Urology, Nanjing Gaochun People's Hospital, Nanjing, China; ^4^Department of Urology, The Fifth Hospital of Xiamen, Xiamen, China

## Abstract

The kidney renal clear cell carcinoma (KIRC) with poor prognosis is the main histological subtype of the renal cell carcinoma, accounting for 80–90% of patients. Currently, the N6-methyladenosine (m6A) epitranscriptional modification draws much attention. The m6A RNA modification, the most plentiful internal modification of mRNAs and noncoding RNAs in the majority of eukaryotes, regulates mRNAs at different levels and is involved in disease occurrence and progression. The GTExPortal and TCGAportal were applied to investigate the METTL14 mRNA expression in different tissues and KIRC stages. The Human Protein Atlas was used to verify the location of METTL14 in KIRC tissues. The main microRNAs (miRNAs) related to KIRC were analyzed using OncoLnc and starBase, while corresponding circular RNAs (circRNAs) interacting with miRNAs were predicted via circBank; then, the METTL14-miRNA-circRNA interaction network was established. The level of methyltransferase-like 14 (METTL14) mRNA was significantly lower in KIRC tissues compared with normal kidney tissues, which was relative to clinical and pathological stages. circRNAs may regulate METTL14 mRNA as miRNAs sponge to affect the KIRC progression. METTL14 mRNA is likely to regulate PTEN mRNA expression via changing its m6A RNA modification level. METTL14 mRNA expression negatively correlated with the KIRC stages and positively correlated with KIRC patients' overall survival, which has great potential to serve as a clinical biomarker in KIRC.

## 1. Introduction

The renal cell carcinoma (RCC) is the most common malignant tumor derived from the kidney. There are approximately 210,000 new cases diagnosed all over the world each year, accounting for 2–3% of all cancers. Currently, the kidney renal clear cell carcinoma (KIRC) is the main histological subtype of RCC and accounts for 80–90% of RCC patients. Yet the prognosis of KIRC is poor [1–3]. At present, the TNM stage and Fuhrman grade are usually used as predictors to assess the risk of patients with KIRC [4, 5]. However, sometimes the outcomes may still be variable for patients with similar clinical features or scores. Meanwhile, a lot of studies indicated that KIRC was heterogeneous in various aspects including clinicopathological, molecular, and cellular heterogeneity. Therefore, it is required and urgent to study the mechanism of KIRC and explore new useful molecular markers for diagnosis and prognosis.

There have been more than 100 kinds of RNA modifications identified in living organisms [6]. Some researches have widely reported certain types of RNA modifications in eukaryotic mRNA, containing N1-methyladenosine (m1A), N6-methyladenosine (m6A), and 5-methylcytosine (m5C), among which m6A was first discovered in the 1970s. Currently, the m6A epitranscriptional modification draws much attention. The m6A RNA modification is the most plentiful internal modification of mRNA and noncoding RNA in the majority of eukaryotes, as well as appreciably clusters around the stop codon and 3′ untranslated region (3′UTR) [7–9]. The physiological importance of m6A RNA modification has been confirmed by its pivotal roles in tissue development and differentiation [10–16]. m6A RNA modification regulates mRNA at different levels, such as structure, maturation, stability, splicing, export, translation, and decay [17]. Moreover, m6A RNA modification is also involved in cell fate decision, cell cycle regulation, cell differentiation, and circadian rhythm maintenance [18]. Numerous RNA-binding proteins (RBPs) are also affected by m6A RNA modification.

In this study, we explored the relationship between methyltransferase-like 14 (METTL14) and KIRC. We used UALCAN to get a heatmap of gene expression related to m6A RNA modification in KIRC and then found that METTL14 mRNA showed a differential expression in KIRC tissues compared with normal tissues. The formation of m6A RNA modification is catalyzed by the RNA methyltransferase complex including methyltransferase-like 3 (METTL3), METTL14, and Wilms tumor 1-associated protein (WTAP), which are the writers of the m6A marks [19]. GTExPortal, TCGAportal, and UALCAN were applied to investigate the expression of METTL14 mRNA in different tissues and KIRC stages.

Additionally, the Human Protein Atlas was used to verify the location of METTL14 in KIRC tissues. MicroRNAs (miRNAs) related to KIRC in PITA, miRmap, and miRanda and corresponding circular RNAs (circRNAs) interacting with miRNAs were analyzed via circBank; then, the METTL14-miRNA-circRNA interaction network was established. Further, both phosphatase and tensin homologue (PTEN) and eukaryotic translation initiation factor 3 subunit A (EIF3A) were found to be positively correlated with METTL14 in KIRC. PTEN is reported as a well-known tumor suppressor gene. EIF3A is a protein encoded by the EIF3A gene in human, which plays crucial roles in regulating translation of a subset of mRNAs, cell cycle progression, and cell proliferation [20]. In this study, PTEN and EIF3A both were found to have a positive correlation with METTL14 in KIRC.

## 2. Materials and Methods

### 2.1. Identification of Genes

The heatmap of gene expressions related to m6A in KIRC was explored in UALCAN (http://ualcan.path.uab.edu/index.html). The expressions of METTL14 mRNA in different tissues were further probed in GTExPortal and UALCAN. In addition, the Human Protein Atlas was used to investigate the location of METTL14 based on pathological slides of KIRC patients. The correlations between expressions of genes were analyzed in GEPIA 2 (http://gepia2.cancer-pku.cn/#index).

### 2.2. The Relationship between METTL14 and KIRC

Via TCGAportal (http://www.tcgaportal.org/), we investigated the expression level of METTL14 mRNA in KIRC stages, mutation types, and corresponding overall survival of KIRC patients. Then, MEXPRESS (http://mexpress.be/) was used to assess the pathological types of KIRC which were related to genders.

### 2.3. The Predictions of the Potential Mechanism

We found the m6A motif base sequence in which METTL14 binds to mRNA and corresponding location using RMBase v2.0 (http://rna.sysu.edu.cn/rmbase/). A total of 9 hub genes related to KIRC and the relationship between them were obtained from TCGAportal. This website was also used to investigate the correlation between METTL14, PTEN, and EIF3A mRNA expressions.

### 2.4. The Identification of Functional Analysis of METTL14

PITA, miRmap, and miRanda, three online tools, were used to explore miRNAs which could bind to METTL14 mRNA. Then, three final sets were merged to pick out common 14 miRNAs (e.g., miR-361, miR-20b, miR-17, miR-106b, and miR-93). OncoLnc (http://www.oncolnc.org/) and starBase (http://starbase.sysu.edu.cn/) were used to further analyze the 14 miRNAs, and finally, the 4 miRNAs were selected. circBank (http://www.circbank.cn/) was applied to find a total of 24 circRNAs interacting with the 4 miRNAs. Subsequently, we constructed the abovementioned miRNAs and circRNAs using Cytoscape to display their interaction network with METTL14 mRNA. Protein interactions among METTL14 and relative proteins were predicted on STRING (http://string-db.org/). GO function analysis for METTL14 mRNA was performed to investigate the molecular function and biological process of METTL14.

## 3. Results

### 3.1. METTL14 mRNA Was Significantly Downregulated in KIRC

**Figure 1:**
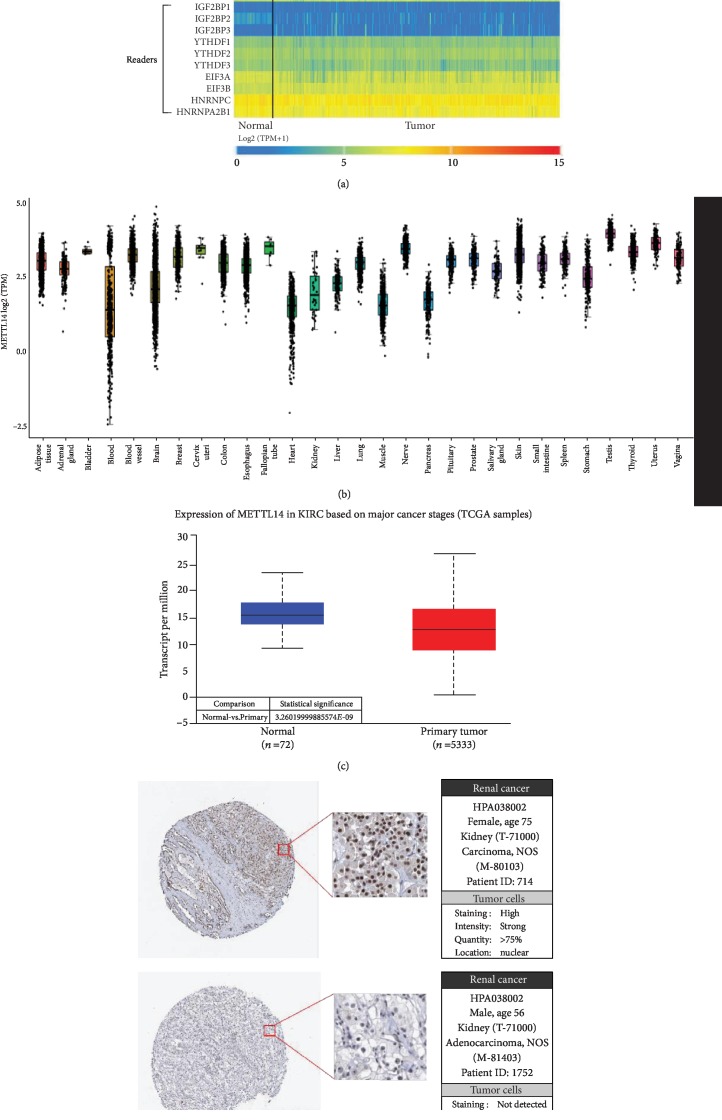
Identification of METTL14 mRNA expression. (a) The heatmap of gene expression related to m6A modification in KIRC. (b) The expression of METTL14 mRNA in GTEx normal tissues. (c) The expression level of METTL14 mRNA in KIRC tissues and adjacent normal kidney tissues. (d) Immunohistochemistry (IHC) analysis of METTL14 expression in KIRC tissues. Representative images were shown.

The heatmap of gene expression related to m6A in KIRC showed that METTL14 mRNA has lower expression in KIRC tissues compared with adjacent normal tissues (Figure 1(a)). The METTL14 mRNA expression in different normal tissues, besides kidney tissues, was further probed to find that METTL14 mRNA has generally higher expression in normal tissues (Figure 1(b)). Additionally, expression of METTL14 mRNA was further confirmed to be significantly downregulated in KIRC tissues compared with adjacent normal kidney tissues (Figure 1(c)). Further, METTL14 was mainly located in the nucleus by immunohistochemistry (Figure 1(d)).

### 3.2. Patients with Lower METTL14 Expression Had a Worse Prognosis

**Figure 2:**
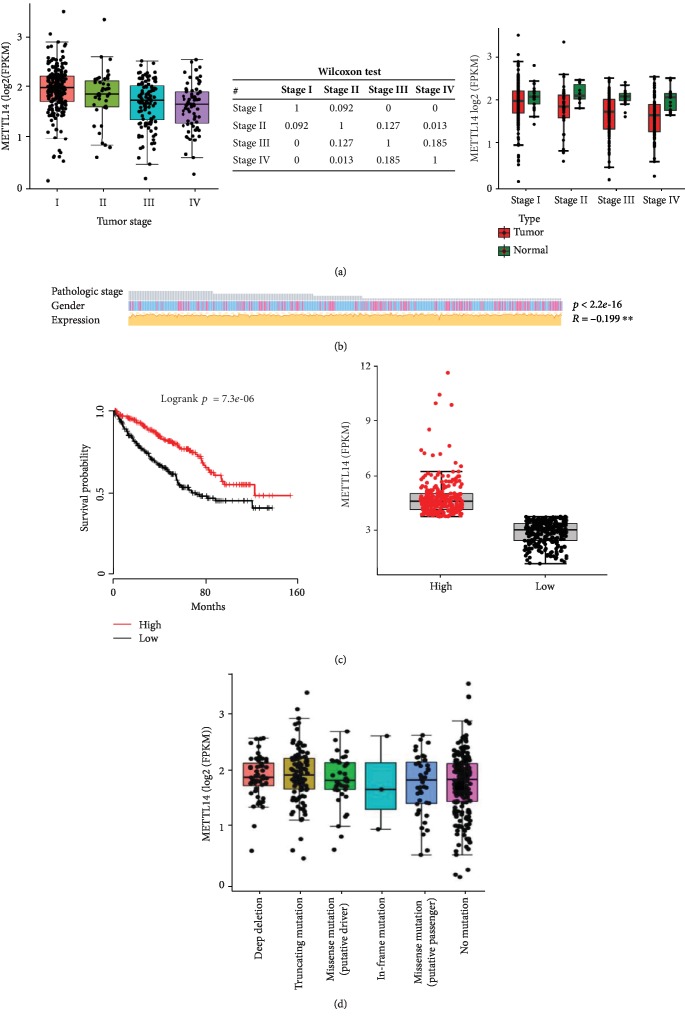
The analysis of METTL14 mRNA in KIRC. (a) The expression of METTL14 mRNA in KIRC stages. (b) The relevance between the METTL14 mRNA expression, pathological stages, and genders. (c) KIRC patients with lower METTL14 mRNA expression level had shorter overall survival. (d) The mutation types of METTL14 mRNA in KIRC.

The METTL14 mRNA expression decreased as the KIRC clinical stage progressed (Figure 2(a)). In addition, the METTL14 mRNA expression has a significantly negative correlation with KIRC pathological stages with male patients more prone to advanced pathological stages by MEXPRESS (Figure 2(b)). Importantly, KIRC patients with lower METTL14 mRNA expression had shorter overall survival (Figure 2(c)). In additionally, the in-frame mutation was the least among mutation types of METTL14 mRNA in KIRC, while the numbers of the other 4 types were close to the quantity of no mutation (Figure 2(d)).

### 3.3. The Potential Mechanism of METTL14 mRNA in KIRC

**Figure 3:**
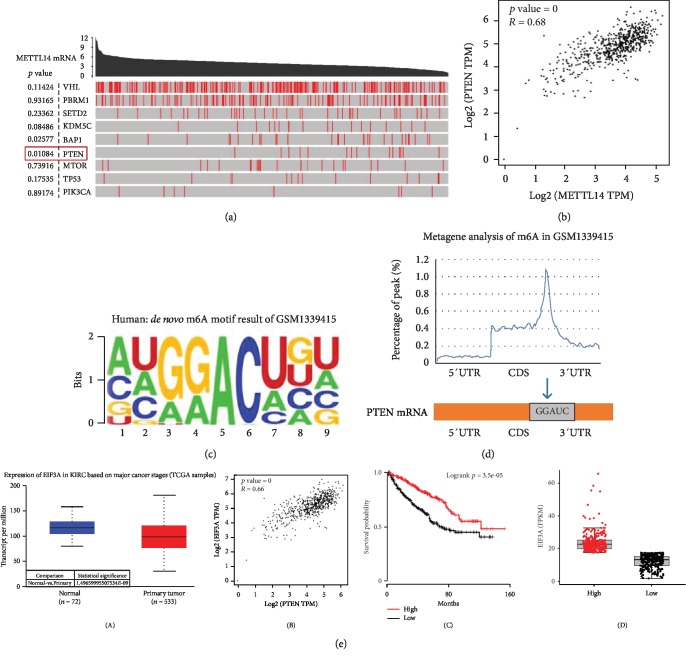
The potential mechanism of METTL14 in KIRC. (a) The relevance between METTL14 mRNA expression and the mutation rate of 9 hub genes in KIRC. (b) The expression of PTEN mRNA and METTL14 mRNA in KIRC had a significantly positive correlation. (c) The m6A motif base sequence in which METTL14 binds to mRNA was more likely to be GGACU (RMBase v2.0). (d) The m6A motif base sequence was located in the junction between CDS and 3′UTR of mRNA with higher probability. The location of GGACU sequence in PTEN mRNA. (e) (A) The expression level of EIF3A mRNA had significantly differential expression in KIRC versus normal tissues. (B) The expression of EIF3A mRNA and PTEN mRNA in KIRC had a significantly positive correlation. (C, D) The TCGA survival analysis for EIF3A.

A total of 9 hub genes related to KIRC were listed, such as VHL, PMRM1, and SETD2. Among these genes, the mutation rate of PTEN mRNA increased as the METTL14 mRNA expression decreased (Figure 3(a)). Further, as the METTL14 mRNA expression increased, the PTEN mRNA expression in KIRC exhibited significant upregulation (Figure 3(b)). METTL14 could act as an m6A writer to affect the stability of some mRNAs and further regulate mRNA expressions [21]. The m6A motif base sequence in which METTL14 binds to mRNA was mainly GGACU, which was located in the junction between the coding sequence (CDS) and 3′-untranslated region (3′-UTR) of mRNAs (Figures 3(c) and 3(d)). EIF3A could act as an m6A reader to participate in m6A RNA modification progression [22]. EIF3A mRNA expression in KIRC tissues was lower than that in normal kidney tissues. A positive correlation between PTEN and METTL14 mRNA in KIRC was observed. We also noticed that as the METTL14 mRNA expression increased, the EIF3A mRNA expression in KIRC exhibited significant upregulation ([Supplementary-material supplementary-material-1]). In addition, patients with lower EIF3A expression had shorter overall survival (Figure 3(e)).

### 3.4. Functional and Pathway Enrichment Analysis

**Figure 4:**
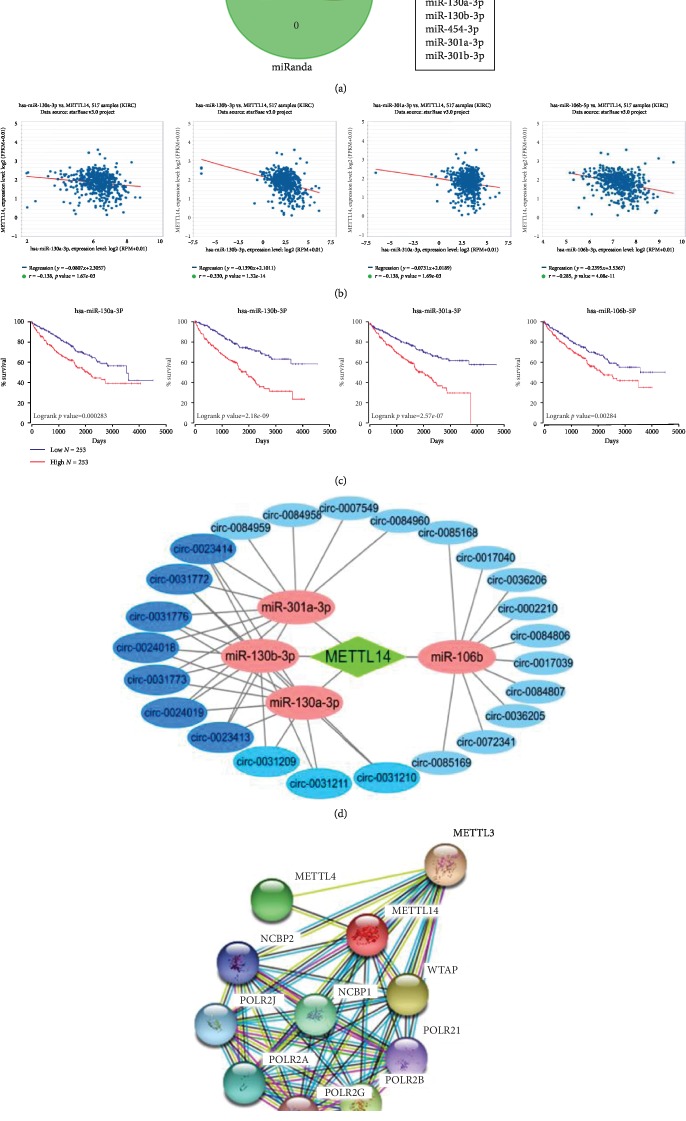
Functional enrichment analyses of METTL14. (a, b) The miRNAs which had potential to bind to METTL14 mRNA. (c) The relevance between the expression of four selected miRNAs and overall survival of KIRC patients. (d) The interaction between METTL14 mRNA, miRNAs, and circRNAs. (e) The protein interaction among METTL14 and relative proteins.

PITA, miRmap, and miRanda were first used to explore some miRNAs binding to METTL14 mRNA separately, and the three final sets were merged to get common 14 miRNAs (e.g., miR-361, miR-20b, miR-17, miR-106b, and miR-93) (Figure 4(a)). The further analysis of 14 miRNAs showed that the expression of miR-130a-3p, miR-130b-3p, miR-106b-5p, and miR-301a-3p had a significantly negative correlation with METTL14 mRNA, respectively, in KIRC (Figure 4(b)). The KIRC patients, who had higher miR-130a-3p, miR-130b-3p, miR-106b-5p, and miR-301a-3p expression level, had shorter overall survival (Figure 4(c)).

A total of 24 circRNAs interacting with the 4 miRNAs were exhibited. Then, an interaction network was constructed based on the abovementioned miRNAs and circRNAs (Figure 4(d)). Protein interaction among METTL14 and relative proteins indicated that METTL14 had potential to play a role by regulating the expression of proteins (Figure 4(e)).

### 3.5. Molecular Function and Biological Process

The results of GO function analysis for proteins interacting with METTL14 demonstrated that the top five molecular functions are DNA-directed RNA polymerase activity, nucleic acid binding, mRNA (2-O-methyladenosine-N6-)-methyltransferase activity, transferase activity, and RNA binding (Table 1), as well as the top five biological processes including 7-methylguanosine mRNA capping, positive regulation of viral transcription, transcription elongation from RNA polymerase II promoter, RNA splicing, and mRNA splicing via the spliceosome (Table 2).

## 4. Discussion

The renal cell carcinoma is a heterogeneous disease, among which the kidney renal clear cell carcinoma was one of the highest incidence subtypes with poor prognosis. Due to limited predictors assessing the risk, a part of KIRC patients might be diagnosed as having inaccurate grade, which would decrease the survival rate of patients with KIRC to some extent. Hence, it is crucial to identify new specific predictors for KIRC.

As a tumor suppressor, PTEN mutation happens frequently in a large number of cancers. The protein encoded by PTEN is phosphatidylinositol-3,4,5-trisphosphate 3-phosphatase, which contains a catalytic domain as well as a tensin-like domain similar to that of the dual-specificity protein tyrosine phosphatases. This protein preferentially dephosphorylates phosphoinositide substrates, which is different from most of the protein tyrosine phosphatases. It negatively regulates intracellular levels of phosphatidylinositol-3,4,5-trisphosphate and functions as a tumor suppressor by negatively regulating the AKT/PKB signaling pathway. METTL14 had a positive correlation with the tumor suppressor gene PTEN, so we speculated that METTL14 exerted a tumor suppressor effect in KIRC by stabilizing PTEN mRNA.

In the previous study, the formation of m6A RNA modification is a reversible process [23]. m6A “writers” with methyltransferase activity consisted of three individual proteins: METTL3, METTL14, and WTAP. FTO and alkB homolog 5 (ALKBH5), m6A demethylases, are called “erasers” [24, 25]. Another protein family is m6A “readers” which can recognize m6A RNA modification to modulate mRNA fate [26]. EIF3A is one of the m6A readers.

In our study, the mRNA expressions of PTEN and EIF3A both had a positive correlation with the expression of METTL14 mRNA in KIRC, suggesting that the interaction between EIF3A and METTL14 may produce synergistic effects and hence jointly regulate KIRC progression. We consider that proteins related to m6A RNA modification have regulatory roles in each other, such as readers in writers and erasers in readers. Additionally, we also doubt whether those proteins are regulated by other modifications like m1A and m5C. The potential mechanism needs further research.

There is another discovery worth talking. The four important functional miRNAs in this study have not been reported yet in KIRC. Given their meaningful characteristics in KIRC, it definitely deserves further exploring and researching.

## 5. Conclusion

Bioinformatics analysis demonstrated that downregulated METTL14 mRNA expression has a significantly negative correlation with the KIRC stages and a positive correlation with overall survival of KIRC patients. Furthermore, circRNAs may regulate METTL14 mRNA as miRNAs sponge to affect the progression of KIRC. METTL14 mRNA is likely to regulate PTEN mRNA expression via changing its m6A RNA modification level. The relationship between KIRC and m6A RNA modification is closely related. In conclusion, METTL14 has great potential to work as a clinical biomarker for KIRC in the future. Further study is needed to explore the value of METTL14 in the stages and prognosis of KIRC.

## Figures and Tables

**Table 1:** GO function analysis for proteins interacting with METTL14 - Molecular function (GO) top 5.

Pathway ID	Pathway description	Count in gene set	False discovery rate
GO:0003899	DNA-directed RNA polymerase activity	5	1.59*e* − 08
GO:0003676	Nucleic acid binding	10	0.000213
GO:0016422	mRNA (2-O-methyladenosine-N6-)-methyltransferase activity	2	0.000216
GO:0016740	Transferase activity	8	0.000459
GO:0003723	RNA binding	7	0.00135

**Table 2:** GO function analysis for proteins interacting with METTL14 - Biological Process (GO) top 5.

Pathway ID	Pathway description	Count in gene set	False discovery rate
GO:0006370	7-Methylguanosine mRNA capping	7	2.17*e* − 14
GO:0050434	Positive regulation of viral transcription	7	6.73*e* − 13
GO:0006368	Transcription elongation from RNA polymerase II promoter	7	4.46*e* − 12
GO:0008380	RNA splicing	9	5.59*e* − 12
GO:0000398	mRNA splicing, via the spliceosome	8	1.7*e* − 11

## Data Availability

The data that support the findings of this study are available from the corresponding authors upon reasonable request.

## Supplementary Materials

Supplementary Materials Figure S1: the correlation between the expressions of EIF3A mRNA and METTL14 mRNA in KIRC.
