# Supplementary figures and images for: Identification of METTL14 in Kidney Renal Clear Cell Carcinoma Using Bioinformatics Analysis
Source: Dis Markers. 2019 Dec 30;2019:5648783. doi: 10.1155/2019/5648783 (PMC6954481; doi:10.1155/2019/5648783)

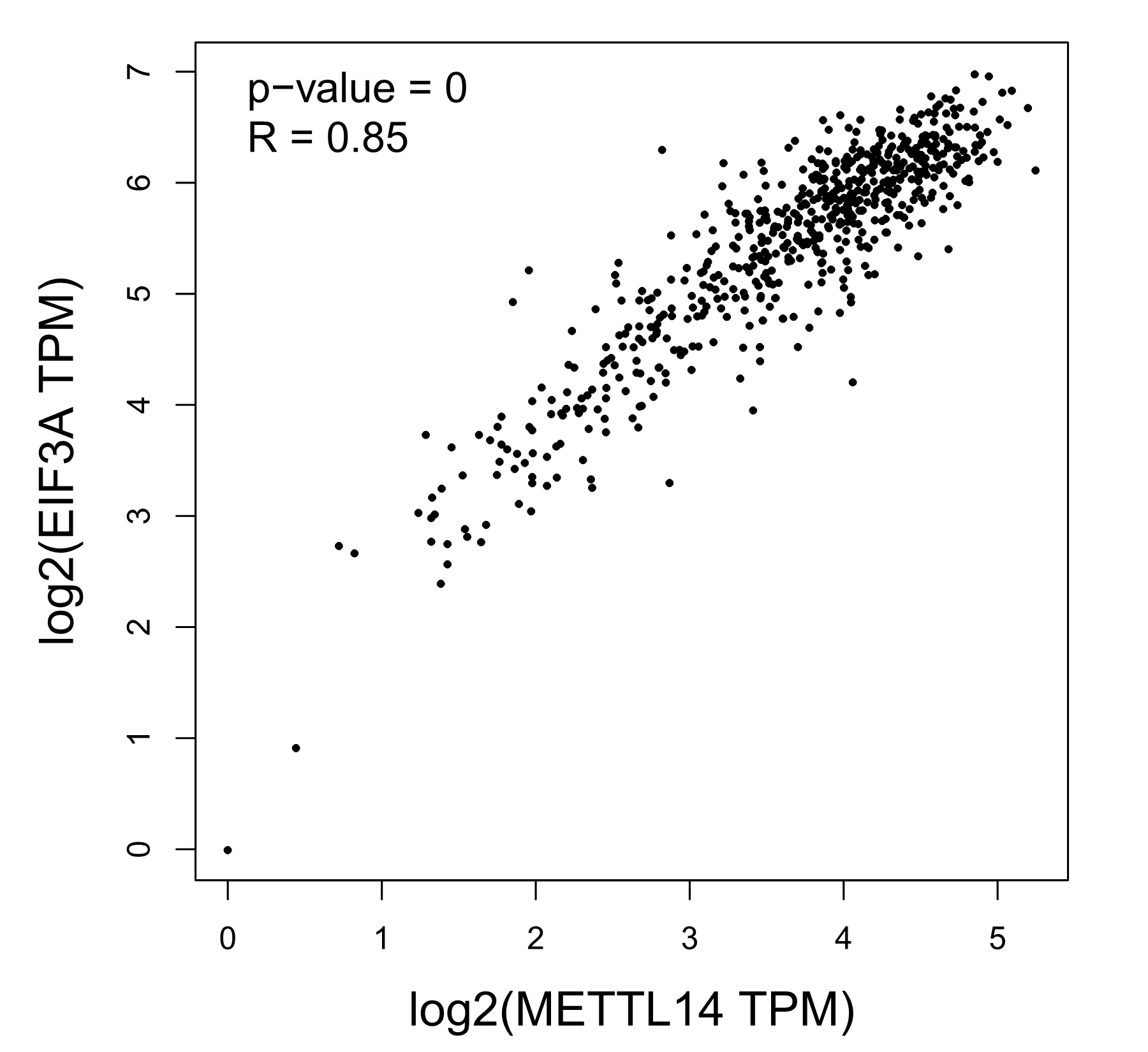

Supplement: Supplementary Materials — Figure S1: the correlation between the expressions of EIF3A mRNA and METTL14 mRNA in KIRC. [file 5648783.f1.jpg]
